# Anemia in Cardiac Surgery - Can Something Bad Get Worse?

**DOI:** 10.21470/1678-9741-2020-0304

**Published:** 2021

**Authors:** Leandro Batisti de Faria, Omar Vilca Mejia, Leonardo Augusto Miana, Luiz Augusto Ferreira Lisboa, Valdano Manuel, Marcelo B. Jatene, Fabio B. Jatene, REPLICCAR Study Group

**Affiliations:** 1 Hospital do Coração (HCor), São Paulo, São Paulo, Brazil.; 2 Cardiovascular Surgery Division, Instituto do Coração do Hospital das Clínicas da Faculdade de Medicina da Universidade de São Paulo (InCor-HCFMUSP), São Paulo, São Paulo, Brazil.; 3 Hospital Samaritano Paulista, São Paulo, SP, Brazil.; 4 Cardio-Thoracic Center, Clínica Girassol, Luanda, Angola.

**Keywords:** Anemia, Cardiac Surgical Procedures, Blood Transfusion, Hematocrit, Cardiopulmonary Bypass, Risk Factors

## Abstract

**Introduction:**

Anemia and blood transfusion are risk factors for morbidity/mortality in patients undergoing cardiac surgery with cardiopulmonary bypass (CPB). The objective of this study is to analyze the association of blood transfusion with morbidity/mortality in patients undergoing coronary artery bypass grafting (CABG) under CPB in the state of São Paulo, Brazil.

**Methods:**

This is a retrospective analysis using the State of São Paulo Registry of Cardiovascular Surgery from November 2013 to August 2014. Blood transfusion was only considered during surgery or within six hours after surgery. Anemia was defined as hematocrit ≤ 37.5%. Patients < 18 years old were excluded. The sample was divided in four groups - Group I (851, no anemia), Group II (200, anemia without blood transfusion), Group III (181, no anemia and transfusion), and Group IV (258, anemia and transfusion).

**Results:**

A total of 1,490 patients were included; 639 (42.9%) were anemic and 439 (29.5%) underwent blood transfusion. Group II showed lower composite morbidity (odds ratio [OR] −0.05; confidence interval [CI] −0.27-0.17; *P*=0.81) than Group III (OR 0.41; CI 0.23-0.59; *P*=0.018) or Group IV (OR 0.54; CI 0.31-0.77; *P*=0.016). Group III was at greater risk of mortality (OR 0.73; CI 0.43-1.03; *P*=0.02) than Group II, which was exposed only to anemia (OR −0.13; CI −0.55-0.29; *P*=0.75), or Group IV (OR 0.29; CI −0.13-0.71; *P*=0.539).

**Conclusion:**

Anemia in patients undergoing CABG with CPB is bad, but blood transfusion can be worse, increasing at least 50% the risk for mortality and/or morbidity.

**Table:** 

Abbreviations, acronyms & symbols	
**BMI**	**= Body mass index**
**CABG**	**= Coronary artery bypass grafting**
**CI**	**= Confidence interval**
**CPB**	**= Cardiopulmonary bypass**
**DM**	**= Diabetes mellitus**
**EuroSCORE**	**= European System for Cardiac Operative Risk Evaluation**
**LITA**	**= Left internal thoracic artery**
**MRS**	**= Myocardial revascularization surgery**
**MV**	**= Mechanical ventilation**
**NYHA**	**= New York Heart Association**
**OR**	**= Odds ratio**
**RA**	**= Radial artery**
**RBC**	**= Red blood cell**
**RITA**	**= Right internal thoracic artery**

## INTRODUCTION

Anemia and blood transfusion are associated with increased morbidity and mortality in heart surgery^[1]^. Although they do not belong to the main risk stratification score variable groups (European System for Cardiac Operative Risk Evaluation [EuroSCORE] and The Society of Thoracic Surgeons Predicted Risk of Mortality), patients who present perioperative anemia are at a higher risk of postoperative renal dysfunction and acute myocardial infarction, longer mechanical ventilation (MV) time, and mortality^[2-4]^.

In addition, lower hematocrit level during cardiopulmonary bypass (CPB) requires blood transfusion, thereby resulting in double risk exposure^[5]^. However, anemia is also associated with numerous other comorbidities and risk factors, such as advanced age, female gender, lower body surface area, ventricular dysfunction, renal failure, and heart failure; therefore, it is difficult to determine whether anemia is essentially a risk factor or is simply a marker of a more serious clinical condition^[6,7]^.

Our study aims to investigate the association of anemia and blood transfusion with morbidity and mortality in patients undergoing heart surgery in the state of São Paulo, Brazil, in three different perioperative management strategies: (1) no blood transfusion; (2) blood transfusion to avoid anemia; and (3) blood transfusion as a treatment of anemia.

## METHODS

This is a retrospective database (State of São Paulo Registry of Cardiovascular Surgery) analysis from November 2013 to August 2014 which enrolled 1,519 patients who underwent cardiac surgery with CPB in the state of São Paulo, Brazil.

We considered only the blood transfusions (red blood cells [RBC]) performed within six hours after surgery to avoid possible confounding bias related to transfusions performed after the occurrence of the morbid events. The anemia for men and women was defined as hematocrit ≤ 37.5 (cutoff) according to the World Health Organization, or WHO^[8]^.

Patients aged < 18 years and those whose preoperative hematocrit level was not available were excluded (29 patients). Of the 1,490 remaining patients included in the statistical analysis, 639 (42.9%) patients were anemic and 439 (29.5%) underwent intraoperative or blood transfusion within six hours after surgery.

For comparison, our cohort was divided into four groups: Group I, (851; 57.1%) patients without anemia (hematocrit > 37.5) who did not receive blood transfusion; Group II, (200; 13.4%) patients with anemia (hematocrit ≤ 37.5), but who did not receive blood transfusion; Group III, (181; 12.1%) patients without anemia (hematocrit ≤ 37.5) who received blood transfusion; and Group IV, (258; 17.3%) patients with anemia (hematocrit ≤ 37.5) who required blood transfusion; double risk exposure.

### Outcomes

The database of the State of São Paulo Registry of Cardiovascular Surgery was prospectively filled according to the definitions of The Society of Thoracic Surgeons National Adult Cardiac Database. The following outcomes were analyzed at discharge and after 30 days: target-organ dysfunction (acute renal failure, acute myocardial infarction, and stroke), infection (operative wound infection, mediastinitis, endocarditis, pneumonia, and sepsis), arrhythmia (atrial fibrillation), surgical re-intervention, postoperative bleeding, cardiogenic shock, and death. We considered composite outcome if any of the abovementioned complications or death took place.

### Statistical Analysis

Categorical variables were expressed as frequencies and percentages, and continuous variables were expressed as averages and standard deviations. Continuous variables with heterogeneous distribution were expressed as medians and confidence intervals (CI) around standard deviations. To compare preoperative and intraoperative characteristics and morbidity and mortality events between the groups, Pearson’s chi-square test, Fisher’s exact test, Student’s *t*-test, and Wilcoxon’s test were used, where indicated. Univariate analysis was used to determine the predictive variables of the composite outcome and in the occurrence of death. Variables found to be significantly (*P*<0.10) related to each of the events after univariate analysis were included in the logistic regression model.

## RESULTS

The baseline and intraoperative data of the four groups are summarized in Table 1. Being an older female with lower body mass index, higher EuroSCORE, and a worse functional class were the common demographic characteristics of anemic patients. They also had a more unstable clinical condition; the majority had emergency hospitalization, preoperative myocardial infarction, and a higher rate of reoperation.

**Table 1:** Baseline characteristics of the 1,490 patients included in the analysis.

Variables		GI (851)	GII (200)	GIII (181)	G IV (258)	*P*-value
Women		234 (27.5%)	92 (46%)	96 (53%)	125 (48.4%)	<0.001
Age (years)		61 (53-68)	64 (54-70)	67 (58-73)	64 (55-72)	<0.001
BMI		27 (24-30)	26 (23-29)	26 (23-29)	26 (23-28)	<0.001
Insulin-dependent DM		90 (10.6%)	40 (20%)	26 (14.4%)	40 (15.5%)	0.002
Hematocrit level		42 (40-45)	36 (34-37)	40 (39-43)	33 (31-35)	<0.001
Ejection fraction		60 (55-65)	60 (53-65)	60 (50-66)	60 (50-66)	0.800
Creatinine clearance		78 (61-96)	65 (47-86)	61 (48-77)	56 (38-76)	<0.001
Atrial fibrillation		120 (14.1%)	23 (11.5%)	18 (9.9%)	39 (15.1%)	0.322
EuroSCORE		1.13 (0.76-1.82)	1.53 (0.9-3.1)	1.95 (1.2-3.75)	2.53 (1.44-5.44)	<0.001
Previous infarction		226 (26.6%)	67 (33.5%)	68 (37.6%)	90 (34.9%)	0.003
NYHA	1	201 (23.6%)	37 (18.5%)	34 (18.8%)	31 (12%)	
	2	325 (38.2%)	83 (41.5%)	65 (35.9%)	85 (32.9%)	
	3	292 (34.3%)	68 (34%)	72 (39.8%)	120 (46.5%)	
	4	33 (3.9%)	12 (6%)	10 (5.5%)	22 (8.5%)	
Previous surgery	0	774 (91%)	178 (89%)	155 (85.6%)	198 (76.7%)	
	1	62 (7.3%)	16 (8%)	19 (10.5%)	36 (14%)	
	2	13 (1.5%)	5 (2.5%)	5 (2.8%)	16 (6.2%)	
	3	2 (0.2%)	1 (0.5%)	2 (1.1%)	6 (2.3%)	
	4	0 (0%)	0 (0%)	0 (0%)	2 (0.8%)	
Hospitalization type	Emergency	2 (0.2%)	3 (1.5%)	2 (1.1%)	4 (1.6%)	
	Elective	625 (73.4%)	136 (68%)	105 (58%)	137 (53.1%)	
	Urgency	224 (26.3%)	61 (30.5%)	74 (40.9%)	117 (45.3%)	
MRS	CPB	472 (55.5%)	118 (59%)	104 (57.5%)	144 (55.8%)	0.812
	LITA	511 (60%)	127 (63.5%)	91 (50.3%)	120 (46.5%)	<0.001
	RITA	76 (8.9%)	13 (6.5%)	2 (1.1%)	10 (3.9%)	<0.001
	RA	38 (4.5%)	7 (3.5%)	2 (1.1%)	8 (3.1%)	0.164
Mitral valve	Plastic	68 (8%)	14 (7%)	8 (4.4%)	15 (5.8%)	0.300
	Bioprosthesis	96 (11.3%)	21 (10.5%)	21 (11.6%)	45 (17.4%)	0.050
	Mechanical prosthesis	33 (3.9%)	8 (4%)	8 (4.4%)	18 (7%)	0.206
Aortic valve	Plastic	14 (1.6%)	1 (0.5%)	1 (0.6%)	7 (2.7%)	0.142
	Bioprosthesis	127 (14.9%)	28 (14%)	45 (24.9%)	61 (23.6%)	<0.001
	Mechanical prosthesis	29 (3.4%)	9 (4.5%)	6 (3.3%)	11 (4.3%)	0.833
Tricuspid valve	Plastic	16 (1.9%)	4 (2%)	5 (2.8%)	16 (6.2%)	0.003
	Bioprosthesis	3 (0.4%)	0 (0%)	1 (0.6%)	0 (0%)	0.379
	Mechanical prosthesis	0 (0%)	0 (0%)	0 (0%)	1 (0.4%)	0.319
Aortic surgery		9 (1.1%)	2 (1%)	8 (4.4%)	3 (1.2%)	0.033
Mortality		37 (4.3%)	17 (8.5%)	26 (14.4%)	45 (17.4%)	<0.001
Morbidity		325 (38.2%)	79 (39.5%)	101 (55.8%)	150 (58.1%)	<0.001

Group I: (851; 57.1%) patients without anemia (hematocrit > 37.5) who did not receive blood transfusion; Group II: (200; 13.4%) patients with anemia (hematocrit ≤ 37.5), but who did not receive blood transfusion; Group III: (181; 12.1%) patients without anemia (hematocrit ≤ 37.5) who received blood transfusion; and Group IV: (258; 17.3%) patients with anemia (hematocrit ≤ 37.5) who required blood transfusion; double risk exposure. BMI=body mass index; CPB=cardiopulmonary bypass; DM=diabetes mellitus; EuroSCORE=European System for Cardiac Operative Risk Evaluation; LITA=left internal thoracic artery; MRS=myocardial revascularization surgery; NYHA=New York Heart Association; RA=radial artery; RITA=right internal thoracic artery

**Fig. 1:**
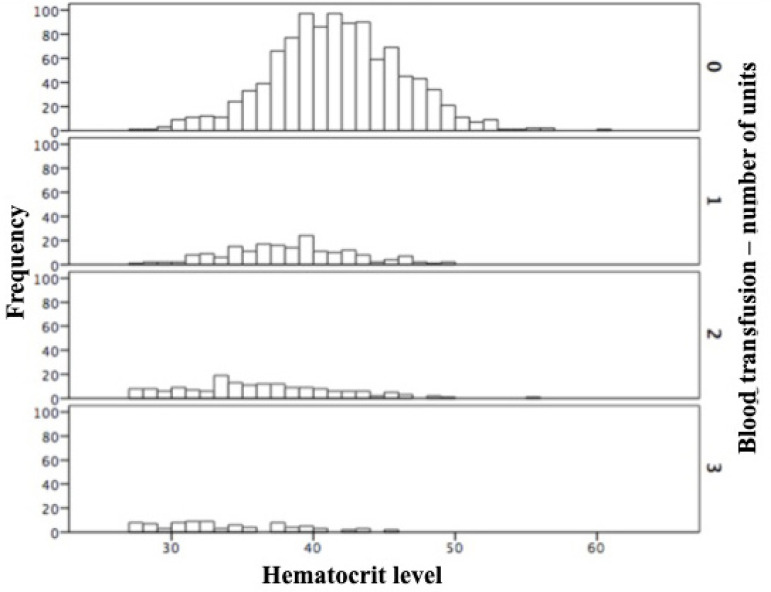
Histogram showing the relationship between preoperative hematocrit level and blood transfusion.

Patients with lower hematocrit required more RBC units during blood transfusion (Figure 1). Of the 1,490 patients, 655 (44%) had at least one of the studied outcomes before 30 days (Table 2).

**Table 2:** Data on discharge and 30-day outcomes.

Variable	No complications	With complications	Complications up to discharge	Complications up to 30 days
Infection	1323 (88.79%)	167 (11.21%)	51 (3.42%)	116 (7.79%)
Pneumonia	1332 (89.4%)	158 (10.6%)	131 (8.79%)	27 (1.81%)
Acute renal failure	1358 (91.14%)	132 (8.86%)	103 (6.91%)	9 (0.6%)
Death	1365 (91.6%)	125 (8.4%)	116 (7.8%)	9 (0.6%)
Arrhythmia	1380 (92.6%)	110 (7.4%)	98 (6.6%)	12 (0.8%)
Atrial fibrillation	1387 (93.1%)	103 (6.9%)	93 (6.24%)	10 (0.6%)
Surgical reoperation	1420 (95.3%)	70 (4.7%)	20 (1.34%)	50 (3.36%)
Low output/cardiogenic shock	1431 (96.04%)	59(4%)	56 (3.76%)	3 (0.2%)
Bleeding	1441 (96.7%)	49 (3.3%)	45 (3.0%)	4 (0.3%)
Sepsis	1442 (96.8%)	48 (3.2%)	37 (2.5%)	11 (0.7%)
Acute myocardium infraction	1465 (98.3%)	25 (1.7%)	22 (1.5%)	3 (0.2%)
Stroke	1468 (98.5%)	22 (1.5%)	14 (0.9%)	8 (0.5%)
Endocarditis	1470 (98.7%)	20 (1.3%)	13 (0.9%)	7 (0.5%)
Mediastinitis	1478 (99.2%)	12 (0.8%)	5 (0.3%)	7 (0.5%)
Systemic inflammatory response syndrome	1481 (99.4%)	9 (0.6%)	9 (0.6%)	0 (0%)

**Fig. 2:**
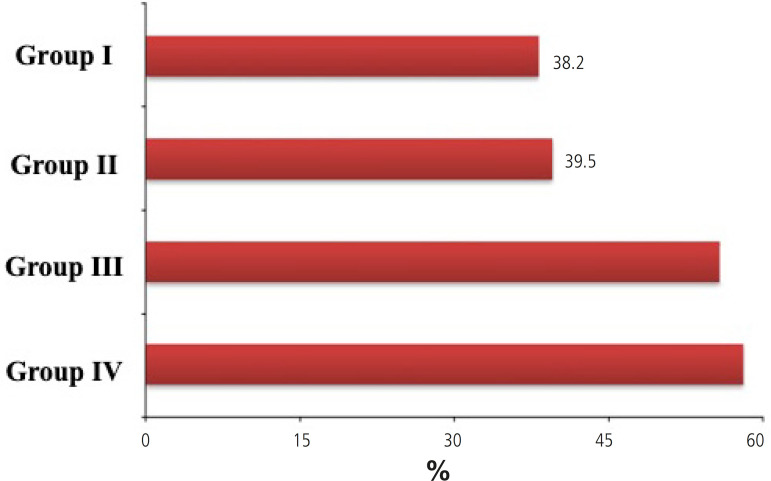
Percentage of combined outcomes in each group.

Unadjusted analysis showed differences in postoperative complications among the groups with more negative exposures associated with worst outcomes. There was no statistically significant difference between the percentage of combined outcomes found in groups not exposed to blood transfusion (*P*>0.05) (Figure 2). However, in the group exposed only to blood transfusion (55.8%) and in the group with double risk exposure (58.1%), the percentage of combined outcomes found was significantly higher (*P*<0.001).

All variables with *P*<0.10 were included in the multiple logistic regression model. Variables in Table 3 were selected as predictors of composite outcome. A patient exposed to blood transfusion had a 1.49-fold greater chance of having a composite outcome (Table 3).

**Table 3:** Predictors of the composite outcome.

	Estimated parameter	Standard error	Odds ratio	95% CI		*P*-value
Blood transfusion	0.401	0.126	1.494	1.166	1.913	0.001
Atrial fibrillation	0.387	0.163	1.472	1.069	2.027	0.018
Age (years)	0.011	0.005	1.011	1.000	1.021	0.04
Creatinine clearance	-0.008	0.002	0.992	0.988	0.997	0.001
EuroSCORE	0.0130	0.026	1.139	1.082	1.199	0.000
Constant	-0.831	0.447				

CI=confidence interval; EuroSCORE=European System for Cardiac Operative Risk Evaluation

**Fig. 3:**
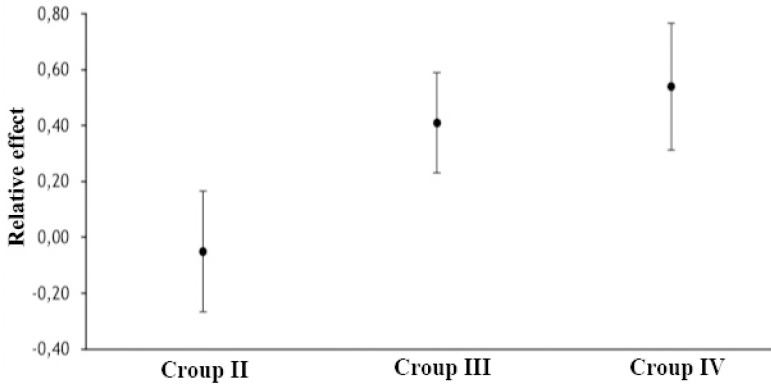
The relative effect of risk exposure on morbidity.

The relative effect of risk exposure to one or both risk factors on morbidity is shown in Figure 3. The Group II (no blood transfusion) (odds ratio [OR] −0.05; 95% CI −0.27-0.17; *P*=0.81) showed lower composite morbidity score than the Group III (blood transfusion) (OR 0.41; 95% CI 0.23-0.59; *P*=0.018) or Group IV (double risk exposure) (OR 0.54; 95% CI 0.31-0.77; *P*=0.016).

**Fig. 4:**
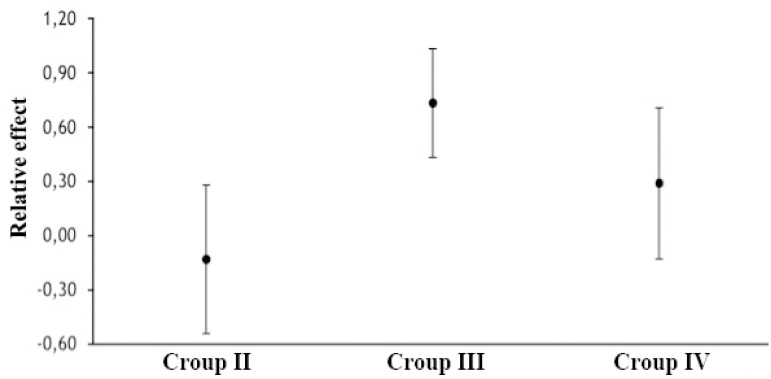
The relative effect of risk exposure on mortality.

The relative effect of risk exposure on mortality is shown in Figure 4. The Group III, exposed to blood transfusion, was at greater risk of mortality (OR 0.73; 95% CI 0.43-1.03; *P*=0.02) than the Group II, exposed only to anemia (OR −0.13; 95% CI −0.55-0.29; *P*=0.75) or Group IV (OR 0.29; 95% CI −0.13-0.71; *P*=0.539).

**Fig. 5:**
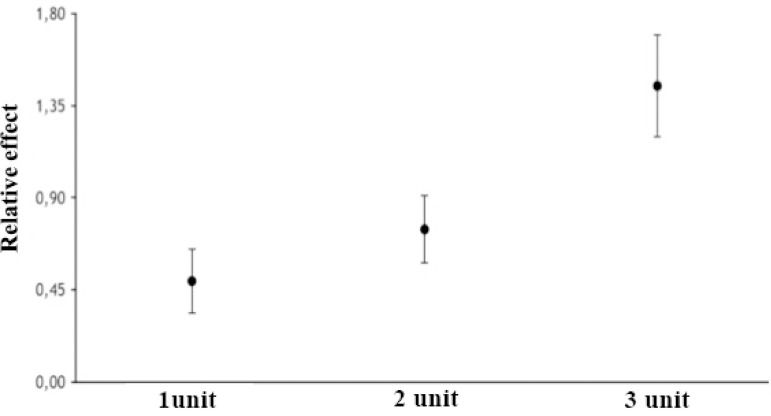
The relative effect on morbidity of the transfused amount of red blood cell units.

Another important finding of this study was the influence of the amount of RBC units on morbidity. Figure 5 shows that the transfusion of three RBC units had a significant effect on morbidity (OR 1.45; 95% CI 1.20-1.70; *P*<0.001).

## DISCUSSION

Anemia is a serious problem in Medicine. Since modern medicine (more than 100 years ago), blood transfusion is performed for management of critically ill patients with hemorrhagic shock and sepsis^[9,10]^. Although transfusion was proved to save lives, it has been involved with complications and adverse events^[1]^.

Preoperative anemia is an independent risk factor for morbidity and mortality in patients undergoing cardiac surgery. A number of studies have demonstrated that anemia can increase the risk for mortality, five-fold higher in some of them, and also major cardiovascular and cerebrovascular events, MV time, and intensive care unit and hospital length of stay compared to non-anemic patients who underwent CABG^[4,6,7,11]^.

Some studies, including randomized clinical trial, with restrictive blood management have demonstrated non-inferiority/superiority compared to liberal strategy regarding patients undergoing cardiac surgery^[12-15]^. The results of the present study were similar to that when the strategy for blood transfusion was restrictive. Liberal strategy was associated with increased risk of morbidity and mortality.

In the present study, it was observed that RBC units transfusion was associated with an increased risk of postoperative cardiac complications, such as severe infection, renal failure, neurological complications, global morbidity, and mortality, both in hospital and up to 30 days after cardiac surgery. These findings are consonant with others in the Literature^[1,12,16,17]^.

This association remained strong even after adjusting for known risk factors associated with adverse outcomes. In each logistic model, RBC unit transfusion was the most reliable indicator associated with the adverse outcome, because it was chosen in 100% of the bootstrap bagging analysis.

A large retrospective study with more than 5,000 patients showed that the RBC transfusion during or after cardiac surgery is associated with short and long-term mortality and its proportional to RBC units received. In addition, they also found association with reoperation, myocardium infarction, arrhythmia, cardiac arrest, stroke, sternal infection, pneumonia, and multi-organ failure^[16]^. A multicenter open-label randomized controlled trial on inferiority, which analyzed adults (5,243 patients) with moderate-to-high EuroSCORE I who underwent cardiac surgery, compared liberal and restrictive strategy and demonstrated that restrictive strategy was not inferior with respect to the composite cause of death, from any cause, myocardium infarction, stroke, renal failure requiring dialysis, and also with less RBC transfusion units^[17]^. It empowers the idea that a liberal RBC strategy increases the risk for morbidity and mortality even in the long-term^[12-15]^.

Although it is unanimous that restrictive strategy is better than liberal, in current literature, the cutoff of tolerated anemia is not well established. In one randomized controlled trial, the cutoff was 8 g/dL, but there are other cutoffs reported in the literature, such as 7.5 g/dL^[12,17]^. There still remains doubt about how much anemia is harmful to the patient and requires transfusion.

Our findings suggest that tolerating anemia (when it is tolerable) is better than to attempt to correct it using RBC unit transfusion, because it exposes the patient to an even bigger risk. These findings are consistent with those by Loor G. et al.^[1]^ and Koch et al.^[5]^, that have shown that the intraoperative treatment for anemia, namely blood transfusions, ironically, also increases risk for morbidity and mortality^[9]^.

Ideally, all patients who will undergo cardiac surgery should be preoperatively clinically optimized to avoid exposure to risk factors that negatively influence outcomes. Anemia is not good, RBC transfusion is bad, but the more units of RBC the patient receives the worse. In the present study, it was well demonstrated; patients who received more RBC units had even worse outcomes. The number of RBC units was also associated with worse long-term survival in recent studies^[15,18]^.

The current study and the most recent evidences support that restrictive strategy is not inferior and liberal strategy is associated with worse outcomes. Therefore, perioperative management of anemia with measures to avoid blood transfusion should be initiated to mitigate transfusion needs.

### Limitations

The retrospective and observational nature of this study is its main limitation. Therefore, there are variables not evaluated in this study that may be a final confounding factor of the analysis and transfusion targets and strategies varied among centers.

However, this is also a multicenter study with a significant number of patients, which confers it external validity, allowing us to generalize our findings to a different population.

## CONCLUSION

RBC transfusion for patients who underwent CABG with CPB was associated with a 50% increased risk of mortality and/or complications. Additional transfusion was associated with an even bigger risk. In patients with anemia that did not receive transfusion, the risk was comparable to non-anemic patients that were not transfused.

**Table:** 

Authors' roles & responsibilities	
LBF	Substantial contributions to the conception and design of the work; drafting the work; final approval of the version to be published
OVM	Substantial contributions to the conception and design of the work; drafting the work; final approval of the version to be published
LAM	Substantial contributions to the conception and design of the work; drafting the work; final approval of the version to be published
LAFL	Revising the work; final approval of the version to be published
VM	Drafting and revising the work; final approval of the version to be published
MBJ	Revising the work; final approval of the version to be published
FBJ	Revising the work; final approval of the version to be published
